# Secretome diversity and quantitative analysis of cellulolytic *Aspergillus fumigatus* Z5 in the presence of different carbon sources

**DOI:** 10.1186/1754-6834-6-149

**Published:** 2013-10-16

**Authors:** Dongyang Liu, Juan Li, Shuang Zhao, Ruifu Zhang, Mengmeng Wang, Youzhi Miao, Yifei Shen, Qirong Shen

**Affiliations:** 1 Jiangsu Key Lab for Organic Solid Waste Utilization, Nanjing Agricultural University, Nanjing 210095, China

**Keywords:** Lignocellulase, Secretome, *Aspergillus fumigatus*, LC-MS/MS, iTRAQ

## Abstract

**Background:**

*Aspergillus fumigatus* Z5 has a strong ability to decompose lignocellulose biomass, and its extracellular protein secretion has been reported in earlier studies employing traditional techniques. However, a comprehensive analysis of its secretion in the presence of different carbon sources is still lacking. The goal of this work was to identify, quantify and compare the secretome of *A. fumigatus* Z5 in the presence of different carbon sources to understand in more details the mechanisms of lignocellulose decomposition by *Aspergillus fumigatus* Z5.

**Results:**

Cellulolytic *A. fumigatus* Z5 was grown in the presence of glucose (Gl), Avicel (Av) and rice straw (RS), and the activities of several lignocellulosic enzymes were determined with chromatometry method. The maximum activities of endoglucanase, exoglucanase, β-glucosidase, laminarinase, lichenase, xylanase and pectin lyase were 12.52, 0.59, 2.30, 2.37, 1.68, 15.02 and 11.40 U·ml^-1^, respectively. A total of 152, 125 and 61 different proteins were identified in the presence of RS, Av and Gl, respectively, and the proteins were functionally divided into glycoside hydrolases, lipases, peptidases, peroxidases, esterases, protein translocating transporters and hypothetical proteins. A total of 49 proteins were iTRAQ-quantified in all the treatments, and the quantification results indicated that most of the cellulases, hemicellulases and glycoside hydrolases were highly upregulated when rice straw and Avicel were used as carbon sources (compared with glucose).

**Conclusions:**

The proteins secreted from *A. fumigatus* Z5 in the present of different carbon source conditions were identified by LC-MS/MS and quantified by iTRAQ-based quantitative proteomics. The results indicated that *A. fumigatus* Z5 could produce considerable cellulose-, hemicellulose-, pectin- and lignin-degrading enzymes that are valuable for the lignocellulosic bioenergy industry.

## Background

Concerns regarding the emission of greenhouse gasses, air pollution due to incomplete combustion of fossil fuel, and shortage of fossil fuels have resulted in an increasing focus on alternative energies such as bioethanol, which can be obtained from lignocellulosics [1]. Lignocellulosic waste composed of cellulose, hemicelluloses, and lignin is one of the largest global carbon sources and is thus considered to be a potential feedstock for the production of biofuel [2]. In the future, biofuel from lignocellulosic biomass will be widely used around the world; additionally, the supply of lignocellulosic biomass from plants or plant-derived materials such as forest and agricultural wastes is inexhaustible [3]. Biofuel is expected to be a renewable resource, and it has the potential to be a major energy source worldwide. However, a large number of cellulosic wastes have been discarded or used inefficiently due to the high cost of utilization processes [4]. Considering these issues, biotransformation has been the subject of intense research in an attempt to develop technologies to convert the abundant quantities of cellulose-rich wastes [5].

The conversion of lignocellulosic biomass into soluble sugars is the primary bottleneck and depends mainly on the production of various efficient lignocellulolytic enzymes [6]. Lignocellulolytic enzymes are a series of enzymes related to lignocellulose degradation, such as cellulases, hemicellulases, pectinases, laccase (Lac), manganese peroxidase (MnP) and lignin peroxidase (LiP). Cellulases, composed of endoglucanases (EGs), cellobiohydrolases (CBHs) and β-glucosidases, are the main enzymes involved in the cellulose degradation process, and they work synergistically to degrade the cellulose fraction [7]. Hemicellulase, produced by a variety of microorganisms during fermentation, is a collective term for a group of enzymes including xylanase, lichenase and laminarinase, which catalyzes the hydrolysis of xylan, lichenin and laminarin, respectively [8]. Pectinase is commonly used in brewing, and pectic enzymes include pectolyase, pectozyme and polygalacturonase. These enzymes break down pectin, a polysaccharide substrate found in plant cell walls [9]. The efficient hydrolysis of lignocellulosic biomass involves the release of long chain polysaccharides and their breakdown into sugars, which requires relayed actions of these enzymes [2,10].

Several strains of fungal and bacterial species are capable of degrading cellulose, hemicellulose and pectin via production of various enzymes and the transport of these enzymes to the outside environment [11]. *Aspergillus* has gained more attention due to its great capacity for secreting different enzymes related to lignocellulose degradation. *Aspergillus niger* NS-2 was able to produce appreciable yields of lignocellulolytic enzymes when wheat bran was used as the substrate in solid state fermentation [12], Matkar [10] studied the optimal production of cellulases by *Aspergillus sydowii* under submerged fermentation. *Aspergillus fumigatus* Z5 (GenBank accession no. GQ337429.1) was isolated from compost and shown to secrete thermostable cellulases under solid state fermentation as indicated by our preliminary studies [13]. However, most recent research has focused on the isolation of cellulolytic strains, the evaluation of lignocellulolytic enzyme production, and purification of the main components [14-16], but very few studies could be documented on the identification and quantification of the secretome of this species. Comprehensive identification and quantification of the secretomes of cellulolytic strains on different carbon sources can be a useful approach for understanding their special and unique enzyme systems. Additionally, such work may help to unravel the lignocellulose hydrolysis mechanism and determine the industrial application of these strains for bioenergy production [17,18].

Although *A. fumigatus* Z5 owned a good capacity in lignocellulose hydrolysis, it should be noted that *A. fumigatus* was a human pathogen, especially for the immunocompromised patients. In order to discover new enzymes and provide a comparison to other *Aspergillus* strains (e.g. *A. niger*) used as industrial hosts, several lignocellulase activities were detected in the liquid medium of *A. fumigatus* Z5, and its secreted extracellular proteins were identified, quantified and compared by a high-throughput, quantitative, iTRAQ-based LC-MS/MS proteomics approach under glucose, Avicel and rice straw.

## Results

### Determination of various degradative extracellular enzyme activities

**Figure 1:**
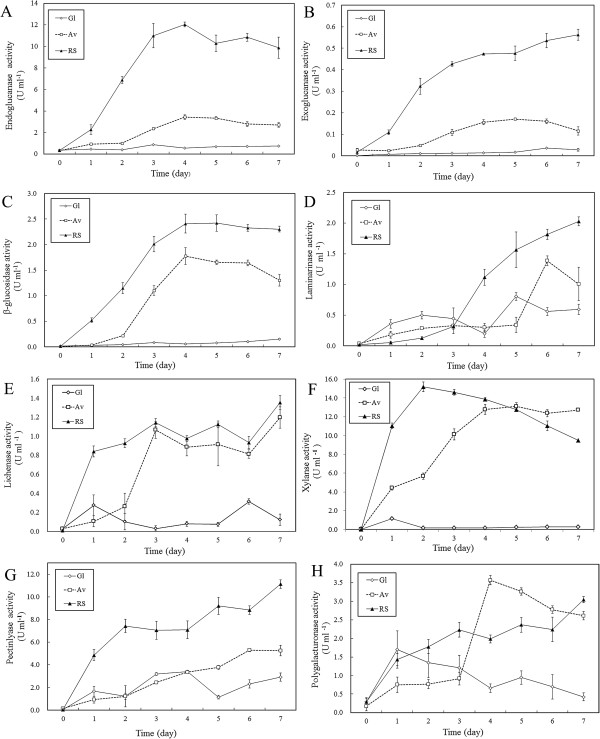
**Activities of extracellular degradative enzymes in the secretome of *A. fumigatus *Z5 in the presence of different carbon sources as determined by spectrophotometric assays.** The results are presented as the mean of three replicates, and bars indicate the standard error of three replicates. Time course profiles of cellulase (i.e., endoglucanase, exoglucanase, and β-glucosidase) production by *A. fumigatus* Z5 on different carbon sources are shown in **A**, **B** and **C**, respectively. Changes in the activities of hemicelluloses such as laminarinase, lichenase, and xylanase as detected by the colorimetric assay are listed in **D**, **E** and **F**, respectively; the production of pectinase by *A. fumigatus* Z5 in the presence of different carbon sources is described in **G** and **H**.

The time courses of various enzymes were determined when glucose, Avicel and rice straw were used as the sole carbon sources, and the results were shown in Figure 1. Endoglucanase activities under the three treatments increased with the incubation time (Figure 1A), and the maximum endoglucanase activity (12.04±0.22 U ml^-1^) of RS was obtained on the 4th day. However, the endoglucanase activity in the presence of Gl was maintained at a low level, and its maximum activity was only 0.87±0.06 U ml^-1^ on the 3rd day. Changes in exoglucanase activities under different carbon source conditions are shown in Figure 1B. Exoglucanase activities increased gradually in the presence of RS, and the highest value (0.56±0.03 U ml^-1^) was obtained at the end of incubation period. The β-glucosidase activities changed significantly in the presence of Av and RS, and the highest activities were both obtained at 4 days (1.78±0.16 U ml^-1^ for Avicel, 2.41±0.19 U ml^-1^ for rice straw). The β-glucosidase activity in the presence of Gl remained at a lower level until the end of the incubation period (Figure 1C).

The activities of hemicellulases, including laminarinase, lichenase and xylanase, were also determined during the incubation period. In the presence of RS, the laminarinase activities increased gradually and reached 2.03±0.07 U ml^-1^ at the end of the incubation period. However, large fluctuations in laminarinase activities were detected in the Av and Gl treatments, and the highest activity in the presence of Av was 1.52±0.08 U ml^-1^ obtained at 6 days (Figure 1D). The lichenase activities were shown in Figure 1E. On the first day, the lowest lichenase activity was obtained with Av, and the activity then increased gradually until the end of the incubation period (1.20±0.11 U ml^-1^). The lichenase activity peak with RS (1.36±0.07 U ml^-1^) was obtained at 7 days. Figure 1F shows a time course of xylanase activities. The xylanase activity with RS was 11.04±0.24 U ml^-1^ on the first day, and the peak (15.19±0.28 U ml^-1^) was reached on the second day, while the xylanase activity in the presence of Av increased gradually, and a peak of 13.10±0.53 U ml^-1^ was obtained at 5 days.

The changes in pectin lyase activities during the cultivation period could be described in Figure 1G. The pectin lyase activity increased gradually in the presence of RS, and the highest activity, i e, 11.14±0.38 U ml^-1^ was obtained at the end of the cultivation period. The change in pectin lyase activity in the presence of Av showed the same trend as that observed with RS, but the highest activity was only 5.26±0.45 U ml^-1^. The polygalacturonase activities were indicated in Figure 1H, and the highest polygalacturonase activity (3.57±0.12 U ml^-1^) appeared at 4 days with Av. In the presence of RS, the polygalacturonase activity increased from 0.29±0.11 U ml^-1^ to 3.05±0.09 U ml^-1^.

In order to comprehensively evaluate the capacity of lignocellulose hydrolysis, ligninolytic enzyme activities (e.g. Lac, MnP and LiP ) and cellobiose dehydrogenase were also measured, and the results were shown in Additional file 1: Figure S1. However, the ligninolytic enzyme and cellobiose dehydrogenase activities were very low, especially when glucose was used as sole carbon sources, and no ligninolytic enzyme activity could be detected in Gl.

### Biomass, protein contents and SDS-PAGE analysis of the supernatants

**Figure 2:**
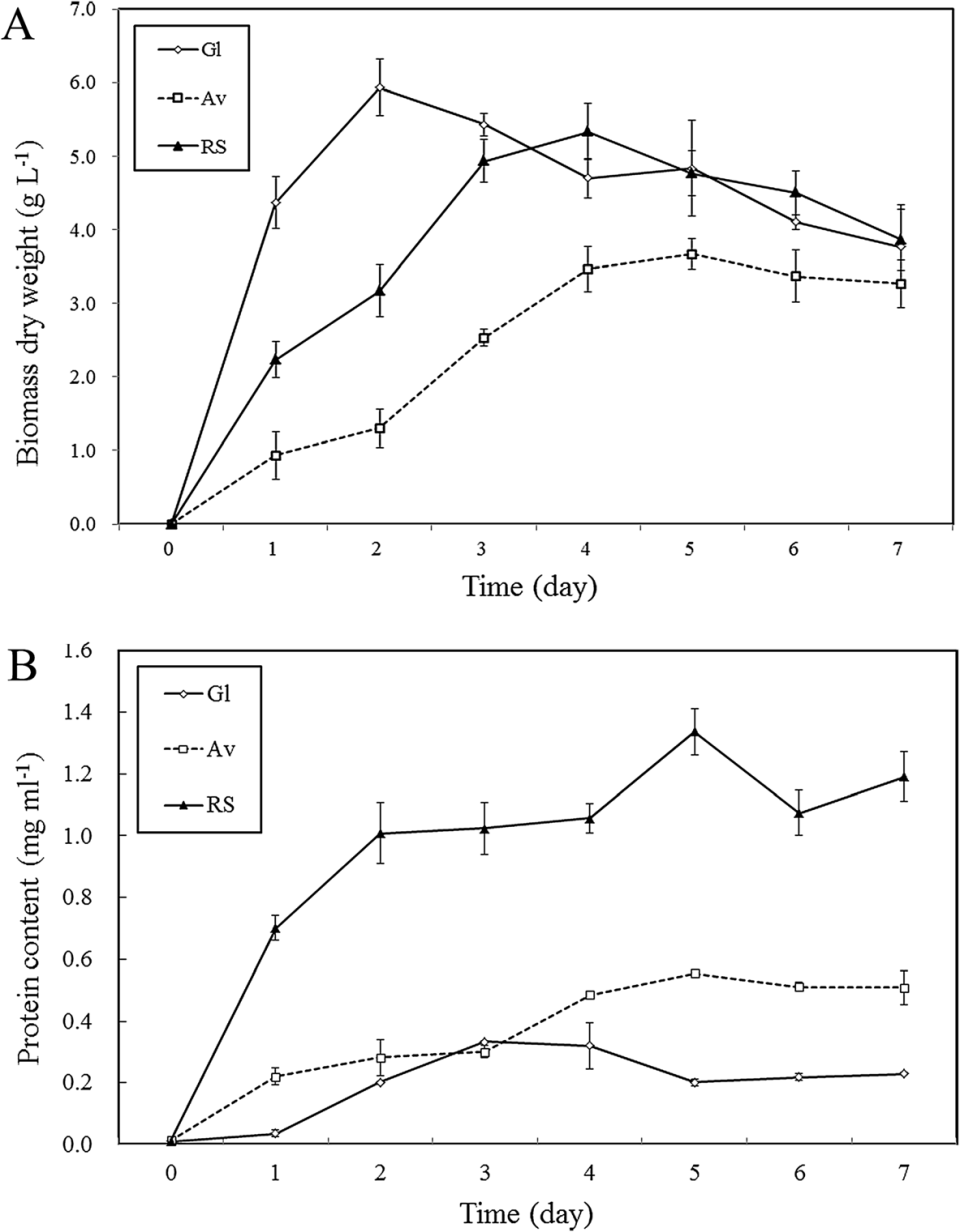
**Biomass and protein contents analysis of *A. fumigatus *Z5 in the present of different carbon sources. (A)** Growth of *A. fumigatus* Z5 induced by different carbon sources. **(B)** Time course of the protein contents of *A. fumigatus* Z5 induced by different carbon sources.

**Figure 3:**
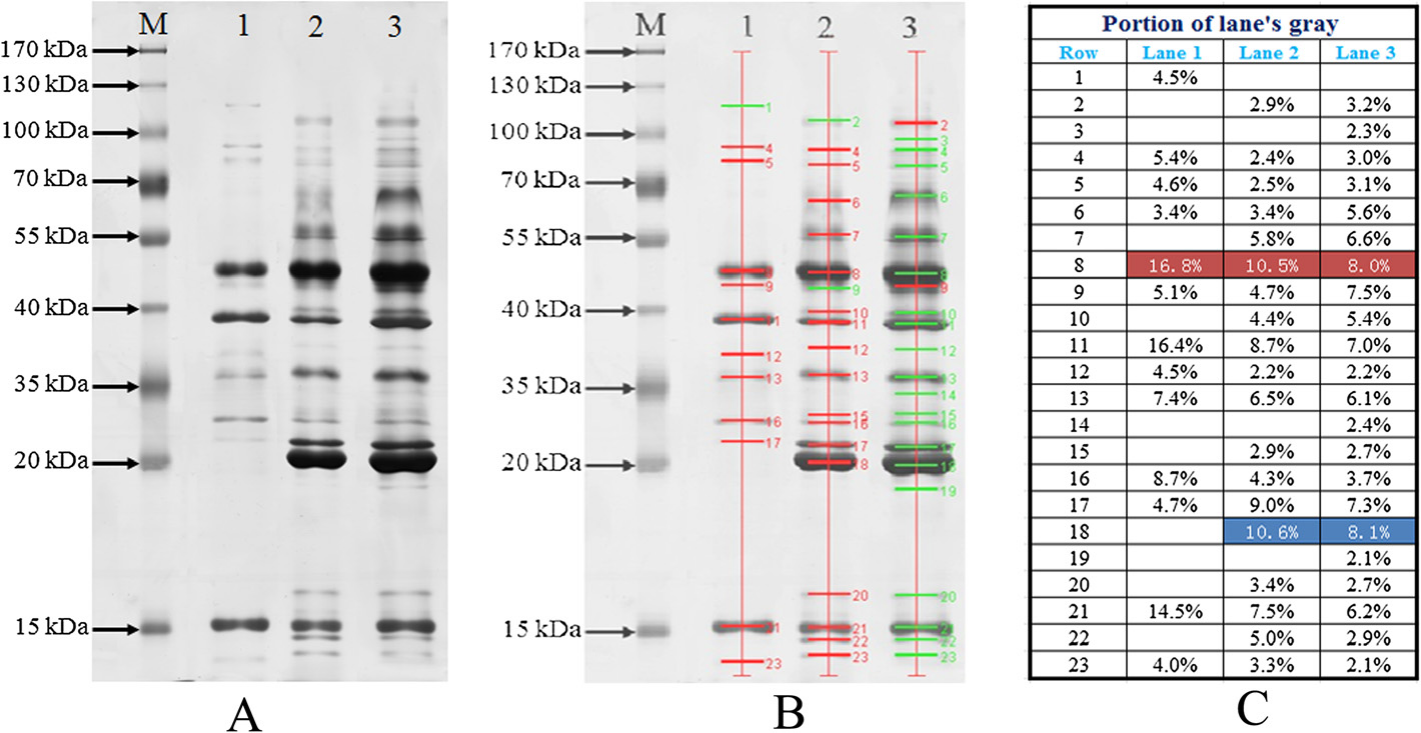
**SDS-PAGE and band analysis using Quantity one (A) SDS-PAGE analysis of the secretomes of *A. fumigatus *Z5 induced by different carbon sources.** Lane M: molecular weight markers. The sizes (kDa) of the proteins in the prestained molecular mass marker are indicated along the left side. Lane 1: The proteins secreted by *A. fumigatus* Z5 when glucose was used as the sole carbon source. Lane 2: The proteins secreted by *A. fumigatus* Z5 when Avicel was used as the sole carbon source. Lane 3: The proteins secreted by *A. fumigatus* Z5 when rice straw was used as the sole carbon source. **(B)** The bands in each lane were selected using Quantity one. There were 12, 19 and 22 bands in lane 1 (Gl), lane 2 (Av) and lane 3 (RS), respectively. **(C)** The proportion of each band in a given lane was calculated and compared across the different treatments.

Figure 2A presented the growth behavior of *A. fumigatus* Z5 on different carbon sources. The cell biomass increased with time and achieved maximum on day 2 in Gl (5.93±0.38 g L^-1^ dw) and day 4 in RS (5.33±0.32 g L^-1^ dw), while corresponding days were 5 for Av (3.67±0.21g L^-1^ dw). The data suggested the growth of *A. fumigatus* Z5 as a function of carbon source. Protein contents in the three treatments were shown in Figure 2B. In the presence of RS, the maximum protein content (1.34±0.07 mg ml^-1^) was obtained at the fifth day, after which it decreased to 1.19±0.09 mg ml^-1^ at the end of incubation period. The maximum protein content (0.55±0.01 mg ml^-1^) was also obtained at the fifth day in the presence of Av. SDS-PAGE analysis of concentrated culture supernatants from different treatments was shown in Figure 3A. Different protein secretomes of *A. fumigatus* Z5 were found depending on the carbon source, and the protein content of each band was also different. The bands in each lane were selected using Quantity One software, and there were twelve, nineteen and twenty two bands in lane 1 (Gl), lane 2 (Av) and lane 3 (RS), respectively (Figure 3B). The proportion of each band in a given lane could be indicated in Figure 3C, and the bands in row 8 (red frame) were the main components in each lane. The percentage of each band in the corresponding lane were 16.8% (Gl), 10.5% (Av) and 8.0% (RS). In the blue frame, the percentage contents of each band in the corresponding lane were 10.6% (Av) and 8.1% (RS).

### Zymogram analysis of extracellular degradative enzymes

**Figure 4:**
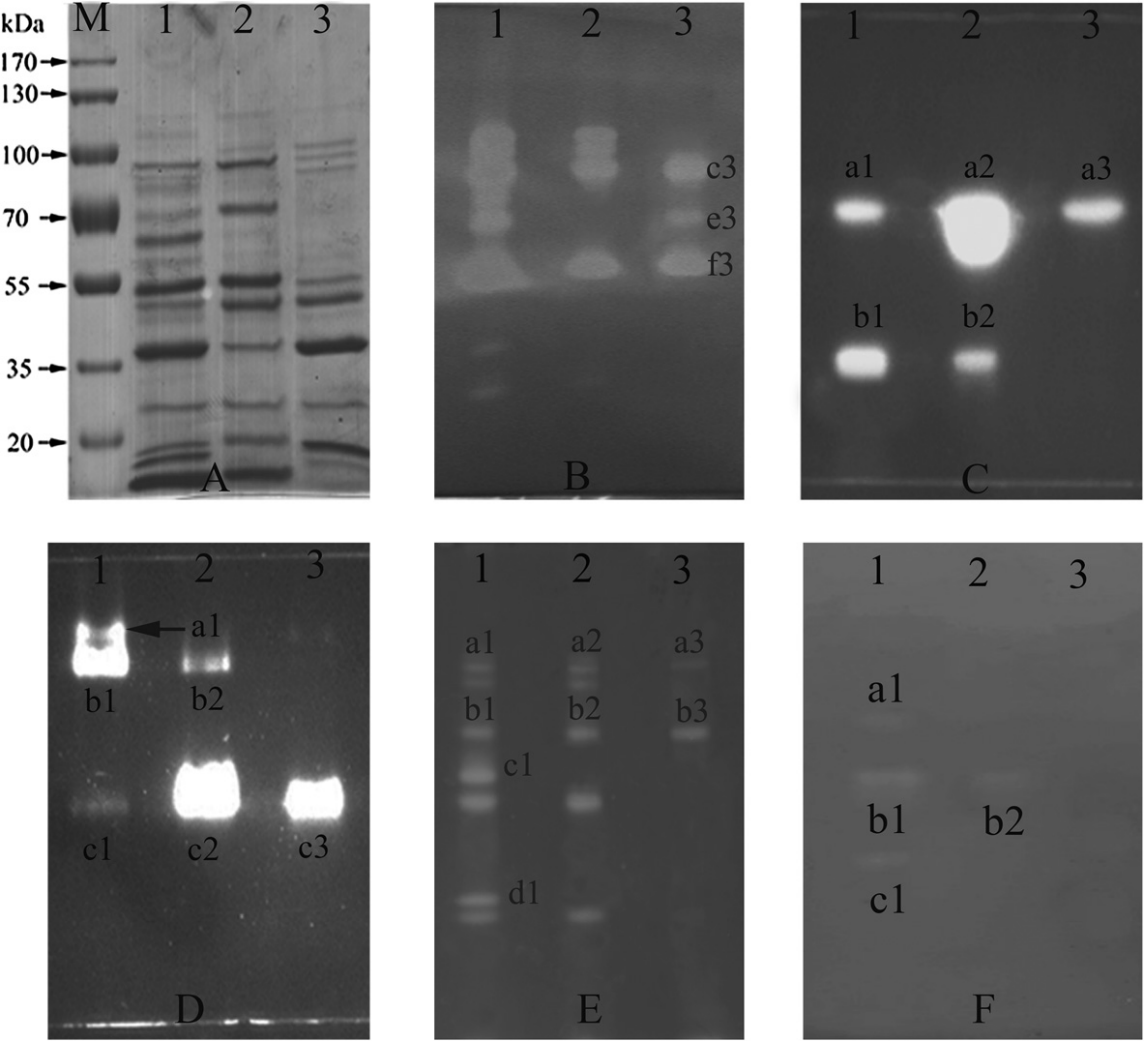
**SDS-PAGE (A) and zymogram analysis of cellulase (B, C, D), xylanase (E) and pectinase (F) produced by *A. fumigatus *Z5.** SDS-PAGE was performed with 0.2% CMC, pNPC, pNPG, xylan and pectin as substrates for endoglucanase, exoglucanase, β-glucosidase, xylanases and pectinases, respectively. Lane M: molecular weight markers; lane 1: zymogram analysis results of the proteins secreted by *A. fumigatus* Z5 in the presence of RS; lane 2: zymogram analysis results of the proteins secreted by *A. fumigatus* Z5 in the presence of Av; lane 3: zymogram analysis results of the proteins secreted by *A. fumigatus* Z5 in the presence of Gl.

The components of the lignocellulose-degrading system of *A. fumigatus* Z5 induced by different carbon sources were detected by zymogram, and the results were shown in Figure 4. Protein bands were examined based on their ability to hydrolyze CMC incorporated into the gel (Figure 4B). After destaining, 8, 5 and 3 major protein bands that were referred to endoglucanase activity were detected in lanes 1, 2 and 3, respectively, and three extra bands presented in the lane of Gl (band c3, e3 and f3). The LC-MS/MS result indicated that glycosyl hydrolase (gi|159121974) was identified in band c3, beta-1,6-glucanase (gi|66845136) was observed in band f3, however no relative proteins were identified in band e3. Results of in-gel exoglucanase analysis was shown in Figure 4C. Two fluorescent bands were detected in lanes 1 and 2; while only one fluorescent band was detected in lane 3, and the LC-MS/MS identification results indicated that cellobiohydrolase (gi|66846140) was in this band. We devised an in-gel activity assay to identify enzymes with β-glucosidase activity, and the analysis results were shown in Figure 4D. In lane 1, three bands showed β-glucosidase activity, and two clear zones were detected in lane 2. However, only one band had the ability to hydrolyze 4-MUG in the Gl lane, and extracellular glycosyl hydrolase/cellulase (gi|66846860) was identified by the LC-MS/MS analysis.

The zymogram analysis of xylanase activity was performed using birchwood xylan as a substrate (Figure 4E). Seven clear zones were obtained in lane 1, and five clear zones were detected in lane 2. however, only two zones were detected in lane 3. The LC-MS/MS result indicated that no xylanase was identified in band a3 and xylosidase/glycosyl hydrolase (gi|66846861) was identified in band b3. The in-gel pectinase activities of the supernatants from different treatments were detected by zymogram analysis, and the results were shown in Figure 4F. In lane 1, three clear zones were detected against the purple background; however, only one zone was detected in lane 2, and no zones were detected in lane 3.

### Identification and quantification of the secretomes from different supernatants

**Figure 5:**
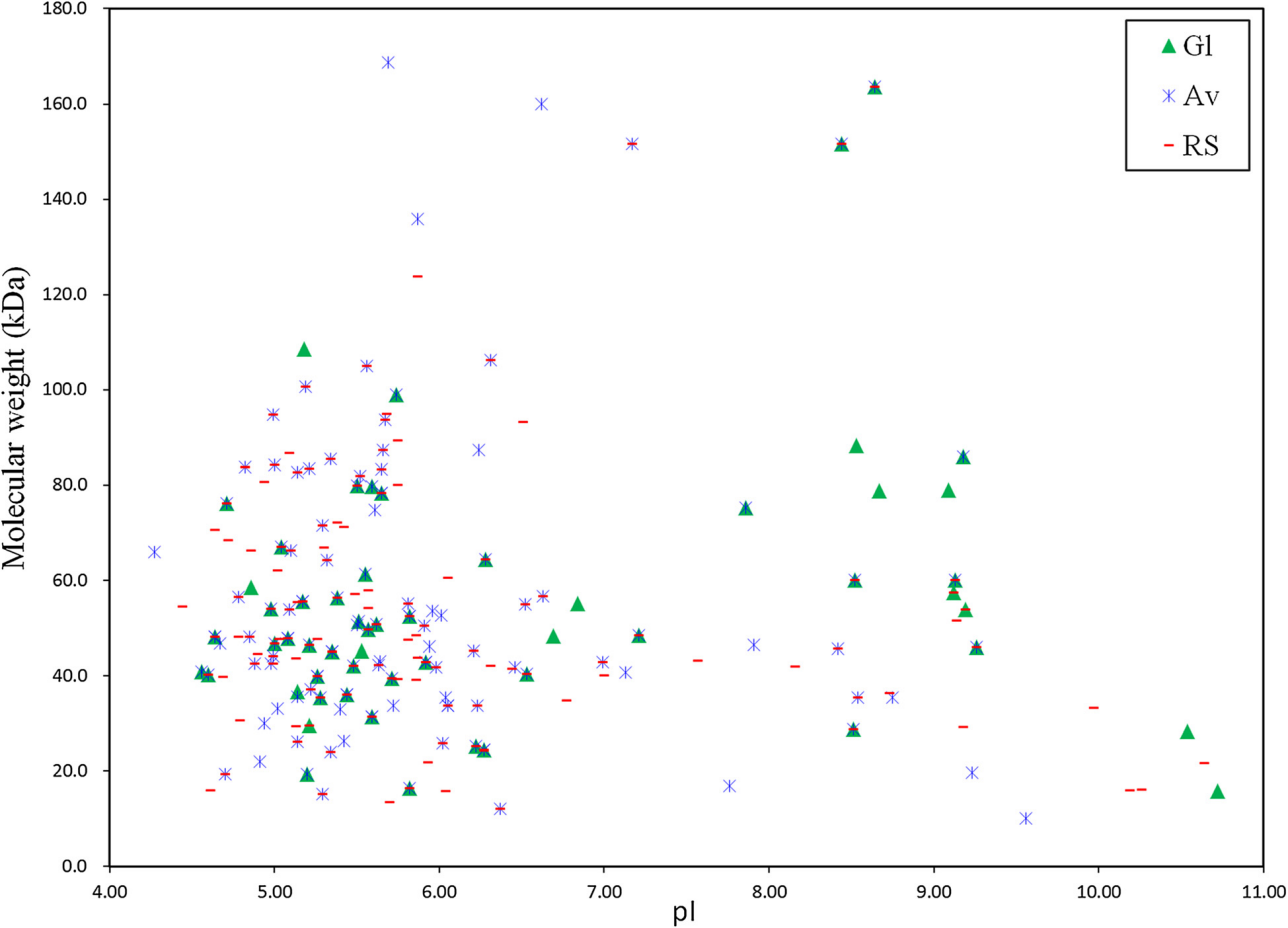
**Distribution of proteins identified by LC-MS/MS as a function of molecular weight and isoelectric point.** Both the molecular and isoelectric point were theoretical value obtained from Compute pI/Mw tool (http://ca.expasy.org/tools/pi_tool.html) according to predicted amino acid sequences.

**Figure 6:**
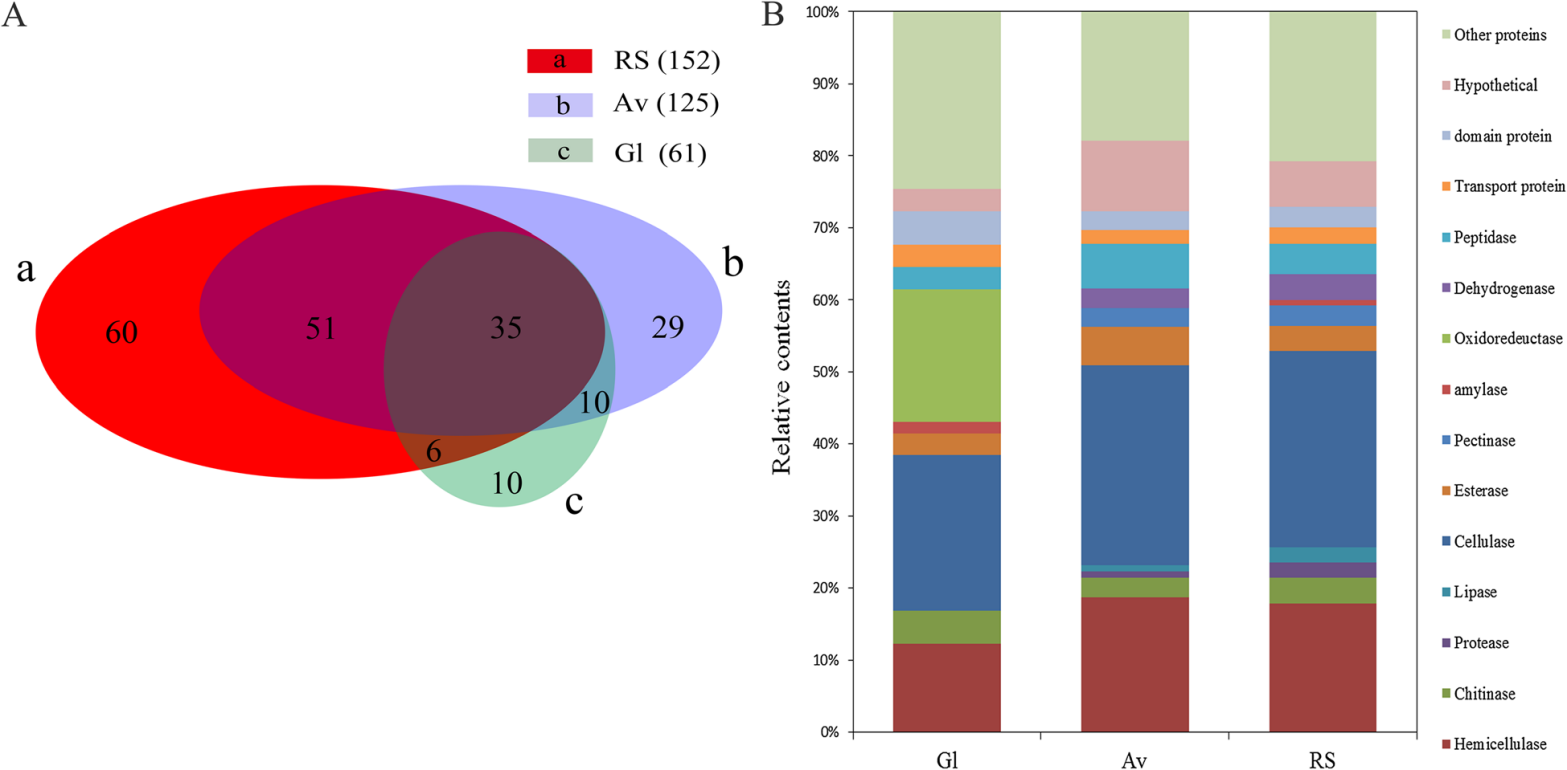
**Identification of the secretome of *A. fumigatus *Z5 in the presence of different carbon sources by LC-MS/MS. (A)** Venn diagram obtained from the comparison of the *A. fumigatus* Z5 proteins induced by different carbon sources as identified by LC-MS/MS. Circle a includes the proteins identified in the supernatant of RS, circle b includes the proteins identified in the supernatant of Av, and circle c includes the proteins identified in supernatant of Gl. **(B)** The relative contents of various proteins identified in the different supernatants.

The proteins that were extracellularly secreted by*A. fumigatu*s Z5 in the presence of different substrates were identified by LC-MS/MS. A total of 221 proteins were identified in the secretome of the different cultures through database searches (NCBInr), out of which thirty-five were present in all the secretome preparations (Additional file 2). The molecular weights of proteins identified by LC-MS/MS along with an isoelectric point curve were shown in Figure 5. The results suggested that the molecular weights of the proteins were in the range of 10–170 kDa, and their isoelectric points ranged between 4.0 and 11.0. The results also indicated that most of the proteins gathered in the acidic side ranging from pI 4–7, which suggested that the secretomes of *A. fumigatus* Z5 were mainly acidic enzyme. Figure 6A showed a Venn diagram illustrating the number of proteins identified in the different cultures. A total of 152 proteins were identified with RS, 125 proteins were identified with Av, and 61 proteins were identified with Gl. The results also indicated that eighty-six proteins were identified in both RS and Av treatments, and forty-five proteins were found in both Av and Gl treatments. There were 35 proteins were identified in all three treatments, and the details were shown in Additional file 3. These LC-MS/MS-identified proteins were functionally classified according to their biological role. As depicted in Figure 6B, the identified proteins were grouped as cellulose-, hemicellulose-, chitin-, protein-, lipid-, pectin-, or starch-degrading enzymes and oxidordeuctase, dehydrogenase, peptidase or transport proteins. The detailed identification results were shown in Additional file 2.

In this study, we performed a comprehensive analysis of the secretory proteins of *A. fumigatus* Z5 with an iTRAQ-based quantitative proteomics approach. The iTRAQ-quantified proteins were functionally classified, and their roles in lignocellulose hydrolysis were listed in Table 1. All proteins identified in the secretome of *A. fumigatus* Z5 that were common in all culture conditions were also listed in Additional file 4. To minimize falsely positive results, a strict cutoff for protein identification was applied with the unused ProtScore ≥1.3, which corresponded to a confidence limit of 95%.

Although this iTRAQ study quantified many cellulose-hydrolyzing enzymes, only fifteen different cellulases and glycosyl hydrolases (including five endoglucanases, three cellobiohydrolases, one β-glucosidase, and six glycosyl hydrolases) that met the cut-off criterion were quantified in all treatments, and the comparative expression levels of the cellulolytic proteins were presented in Table 1. When rice straw was used as the sole carbon source, all the identified cellulolytic proteins were upregulated except GH17 β-1, 3-endoglucanase EglC (gi|66845978); however, GH17 beta-1 and 3-endoglucanase EglC were upregulated when *A. fumigatus* Z5 was cultivated with Avicel compared with their levels under cultivation with glucose. GH61 endo-1,4-beta-glucanase (gi|66846336) and GH12 endoglucanase (gi|66846525) were downregulated when *A. fumigatus* Z5 was cultivated with Avicel, and the iTRAQ ratios were 0.09±0.02 and 0.14±0.01, respectively. When rice and Avicel were used as carbon sources, GH2 endoglucanase (gi|66848676) was significantly upregulated with iTRAQ ratios of 0.33±0.07 and 0.10±0.00, respectively. In this study, when different types of carbon sources were used for cultivation, three cellobiohydrolases were expressed and quantified by iTRAQ. The GH6 cellobiohydrolase (gi|66846140) was identified in the presence of RS, Av and Gl, and the iTRAQ ratios were 0.19±0.02, 0.10±0.01, and 0.09±0.01, respectively; the GH7 cellobiohydrolase (gi|74670999) was also identified in the presence of RS, Av and Gl, and the iTRAQ ratio of GH7 cellobiohydrolase with RS was 4 times higher than that with Gl; however, it was only 1.5 times higher than that with Avicel. GH7 1,4-beta-D-glucan-cellobiohydrolase (gi|74670557) was also identified and iTRAQ-quantified under all treatments. Only one β-glucosidase was iTRAQ-quantified in all treatments: the cellobiose-hydrolyzing GH3 β-glucosidase (gi|66850743), which was upregulated in both RS and Av, and the 116/119 and 117/119 ratios were 7.00 and 4.33, respectively. In addition to cellulases, six proteins belong to glycoside hydrolase were also iTRAQ-quantified, and the secretion signal analysis results indicated that all of these six proteins had signal peptides.

**Table 1:** Functional classification and quantitation of the same proteins identified in different treatments

**Accession no.**	**Name of proteins**	**LIBER number peptides**	**num unique peps**	**tot indep spectra**	**percent share of spectrum id's**	**iTRAQ Ratio**			**116/119**	**117/119**	**Family**	**Signal P**
						**116**	**117**	**119**				
**Cellulase**												
gi|66844580	glycosyl hydrolase family 43 protein	4	1	7	0.21	0.16±0.03	0.11±0.01	0.03±0.01	5.33	3.67	GH 43 (PF04616)	Y
gi|66845136	beta-1,6-glucanase Neg1	2	1	2	0.13	0.17±0.02	0.47±0.02	0.10±0	1.70	4.70	GH 30 (PF02055)	Y
gi|66845978	beta-1,3-endoglucanase EglC	25	2	29	1.89	0.04±0.01	0.63±0.03	0.11±0.01	0.36	5.73	GH 17 (PF00332)	Y
gi|66846140	cellobiohydrolase	10	1	12	0.76	0.19±0.02	0.10±0.01	0.09±0.01	2.11	1.11	GH 6 (PF01341)	Y
gi|66846336	endo-1,4-beta-glucanase	10	3	10	0.64	0.24±0.01	0.09±0.02	0.13±0.02	1.85	0.69	GH 61 (PF03443)	Y
gi|66846525	endoglucanase	6	2	6	0.39	0.27±0.03	0.14±0.01	0.19±0.04	1.42	0.74	GH 12 (PF01670)	Y
gi|66846526	beta-D-glucoside glucohydrolase	13	2	15	0.97	0.28±0.01	0.16±0.01	0.11±0.01	2.55	1.45	GH 3 (PF00933)	Y
gi|66846837	glucan 1,4-alpha-glucosidase	3	2	1	0.13	0.31±0.04	0.18±0.09	0.05±0.01	6.20	3.60	GH 15 (PF00723)	Y
gi|66846860	extracellular glycosyl hydrolase	1	1	1	0.09	0.17±0.03	0.15±0.01	0.07±0.01	2.43	2.14	GH 62 (PF03664)	Y
gi|66848676	endoglucanase	2	1	2	0.13	0.33±0.07	0.10±0	0.03±0	11.00	3.33	GH 5 (PF00150)	Y
gi|66849674	endoglucanase	58	15	109	6.98	0.31±0.01	0.12±0	0.04±0	7.75	3.00	GH 61 (PM03443)	Y
gi|66850620	extracellular cell wall glucanase	1	1	1	0.07	0.2±0.04	0.55±0.07	0.06±0	3.33	9.17	GH 16 (PF00722)	Y
gi|66850743	beta-glucosidase	1	2	1	0.15	0.42±0	0.26±0.02	0.06±0	7.00	4.33	GH 3 (PF01915)	Y
gi|74670557	1,4-beta-D-glucan-cellobiohydrolyase	21	12	100	7.45	0.28±0.01	0.11±0.01	0.06±0.01	4.67	1.83	GH 7 (PF00840)	Y
gi|74670999	cellobiohydrolase	17	12	35	3.22	0.30±0.01	0.09±0.01	0.06±0.01	5.00	1.50	GH 7 (PF00840)	Y
**Hemicellulase**												
gi|66845449	alpha-galactosidase	2	2	3	0.12	0.25±0.01	0.13±0.11	0.11±0.08	2.27	1.18	Melibiase (PF02065)	N
gi|66845983	endo-1,4-beta-xylanase (XlnA)	31	9	49	3.16	0.34±0.01	0.07±0	0.02±0.01	17.00	3.50	GH 11 (PF00457)	Y
gi|66846833	arabinosidase	1	2	2	0.08	0.44±0.09	0.10±0	0.03±0	14.67	3.33	GH 43 (PF04616)	N
gi|66846861	xylosidase	1	2	4	0.20	0.72±0	0.21±0	0.01±0	72.00	21.00	GH 43 (PF04616)	Y
gi|66848870	endo-1,4-beta-xylanase	21	7	35	2.27	0.35±0.01	0.08±0.01	0.05±0.01	7.00	1.60	GH 10 ( PF00331 )	Y
gi|66850460	mannosidase MsdS	1	2	6	0.09	0.44±0.07	0.06±0.01	0.13±0.02	3.38	0.46	GH 47 ( PF01532)	Y
gi|66851740	endo-1,4-beta-xylanase	2	2	3	0.12	0.35±0.02	0.13±0.05	0.11±0.08	3.18	1.18	GH 10 ( PF00331)	Y
gi|70981394	extracellular arabinanase	4	3	4	0.26	0.27±0.03	0.24±0.01	0.11±0.06	2.45	2.18	GH 43 (PF04616)	Y
gi|70994060	extracellular endo-1,4-beta-xylanase	17	10	26	1.62	0.31±0.01	0.08±0	0.06±0.01	5.17	1.33	GH 10 (PF00331)	Y
gi|74668470	endo-1,4-beta-xylanase	5	3	5	0.23	0.28±0.05	0.24±0.01	0.13±0.06	2.15	1.85	GH 11 (PF00457)	Y
**Chitinase**												
gi|129556953	class V chitinase	1	1	1	0.09	0.27±0.03	0.10±0.01	0.35±0.07	0.77	0.29	GH18 (PF00704)	Y
gi|129558344	class V chitinase	6	8	14	0.86	0.24±0.01	0.26±0.01	0.1±0.02	2.40	2.60	GH 18 (PF00704)	Y
gi|66846625	class III chitinase	5	5	5	0.30	0.19±0.04	0.17±0.06	0.15±0.06	1.27	1.13	GH 18 (PF00704)	Y
gi|70983075	class V chitinase ChiB1	72	23	159	10.35	0.27±0.01	0.09±0	0.11±0.01	2.45	0.82	GH 18 (PF00704)	Y
**Phosphatase, lipase, pectinase, lignin degrading proteins, esterase and protease**												
gi|129557040	Ser/Thr protein phosphatase family protein	1	2	2	0.09	0.49±0.08	0.06±0.01	0.15±0.02	3.27	0.40	Metallophos (PF00149)	N
gi|129558573	esterase	1	2	3	0.18	0.07±0	0.26±0.02	0.55±0.05	0.13	0.47	-	Y
gi|66845766	extracellular lipase	37	7	64	4.12	0.07±0.01	0.34±0.01	0.35±0.02	0.20	0.97	Lipase 3 (PF01764)	Y
gi|66846844	pectate lyase	1	1	1	0.07	0.73±0.04	0.15±0.01	0.25±0.04	2.92	0.60	Pec_lyase_C (PF00544)	Y
gi|66849090	metalloprotease	10	4	13	0.84	0.38±0.07	0.1±0.03	0.07±0.04	5.43	1.43	Peptidase_M35 (MF02102)	Y
gi|66853735	cellobiose dehydrogenase	10	3	12	0.79	0.16±0.02	0.41±0.03	0.19±0.02	0.84	2.16	GMC_oxred_N (PF00732)	Y
gi|70986104	CAT1 mycelial catalase Cat1	8	5	10	0.60	0.21±0.02	0.09±0.01	0.25±0.02	0.84	0.36	Catalase (PF00199)	N
gi|70997966	Cu, Zn superoxide dismutase	4	1	4	0.26	0.18±0.03	0.19±0.01	0.18±0.01	1.00	1.06	Sod_Cu (PF00080)	N
**Permease, reductase, helicase and Transporter**												
gi|66844481	high affinity methionine permease	2	1	2	0.13	0.28±0.01	0.19±0.02	0.17±0.02	1.65	1.12	AA_permease2 ( PF13520)	N
gi|66848496	thioredoxin reductase GliT	2	4	4	0.23	0.14±0.08	0.37±0.06	0.29±0.01	0.48	1.28	Pyr_redox_2 (PF07992)	N
gi|66848977	telomere-associated RecQ helicase	31	3	52	3.16	0.01±0	0.47±0	0.46±0.01	0.02	1.02	DUF3505 (PF12013)	N
gi|70982865	MFS transporter	8	3	47	2.40	0.26±0	0.29±0.01	0.15±0	1.73	1.93	MFS_1 (PF07690)	N
**Other proteins**												
gi|129555482	cytochrome P450	5	2	5	0.29	0.11±0.03	0.27±0.03	0.31±0.10	0.35	0.87	p450 (PF00067)	N
gi|129555486	secreted antimicrobial peptide	3	2	3	0.16	0.10±0.04	0.51±0.09	0.20±0.01	0.50	2.55	MiAMP1 (PF09117)	Y
gi|146324637	hybrid PKS-NRPS enzyme	2	2	3	0.13	0.18±0.02	0.16±0.06	0.32±0.02	0.56	0.50	Acyl_transf_1 (PF00698)	N
gi|66847207	HECT domain protein	5	3	8	0.51	0.29±0.03	0.12±0.01	0.15±0.02	1.93	0.80	HECT (PF00632)	N
gi|66847547	C6 finger domain protein	2	1	2	0.13	0.02±0	0.51±0	0.39±0.02	0.05	1.31	Zn_clus (PF00172)	N
gi|66851935	aldose 1-epimerase	2	2	13	0.11	0.26±0.04	0.16±0.06	0.32±0.07	0.81	0.50	*Aldose-epim* (PF01263)	N
gi|66852539	glutaminase GtaA	2	2	3	0.18	0.40±0.01	0.03±0	0.07±0	5.71	0.43	DUF1793 (PF08760)	Y
gi|66853400	translation initiation factor eIF-2B	49	1	64	3.95	0.30±0	0.27±0	0.09±0	3.33	3.00	*W2* (PF02020)	N

The peptides from RS, Av and Gl were labeled with the iTRAQ tags 116, 117 and 119, respectively.

This study identified and iTRAQ-quantified ten different hemicellulases, including xylanases, xylosidases, mannosidases, arabinases, and galactosidases, under all three treatments. The comparative expression levels of iTRAQ-quantified hemicellulose-degrading proteins could be found in Table 1, and the iTRAQ quantification results showed that the expression levels of all the hemicellulases varied depending on the type of carbon source. In this study, five different endo-1,4-beta-xylanases (gi|66845983, gi|66848870, gi|66851740, gi|70994060, gi|74668470) were iTRAQ-quantified in all treatments, all of which were significantly expressed in the presence of RS relative to Gl. However, compared with their levels in the presence of rice straw, the contents of GH11 xylanase (gi|66845983), GH10 xylanase (gi|66848870) and GH10 xylanase (gi|70994060) were low when Avicel was used for strain culture. GH43 xylosidase (gi|66846861) was significantly produced by *A. fumigatus* Z5 in the presence of RS (0.72) and Av (0.21) when compared to Gl (0.01). GH47 mannosidase MsdS (gi|66850460), which hydrolyzes mannose in substrates, was identified and quantified in all treatments, and the iTRAQ ratio in the presence of RS was 0.44±0.07. However, the lowest iTRAQ ratio (0.06±0.01) was obtained with Av. iTRAQ quantification showed significant upregulation of the hemicellulases, indicating the lignocellulose-hydrolyzing potential of *A. fumigatus* Z5. Of the identified hemicellulases, GH11 alpha-galactosidase (gi|66845449) and GH43 arabinosidase (gi|66846833) lacked secretion signals.

The iTRAQ data demonstrated the secretion of different chitinases by *A. fumigatus* Z5 under different culture conditions, and four different glycosyl hydrolase family 18 chitinases were iTRAQ quantified. The iTRAQ ratio of class V chitinase (gi|129558344) was 0.24±0.01 in the presence of RS, which was 2.4 times higher than that observed with Gl, and the quantity of this chitinase was also higher (0.26±0.01) in the presence of Av compared with Gl. However, another class V chitinase (gi|129556953) showed the opposite result, and the highest iTRAQ ratio (0.35±0.07) was obtained with Gl.

In addition to cellulases, hemicellulases and chitinases, we additionally quantified several other enzymes via iTRAQ. Pectin depolymerizing pectate lyase (gi|66846844) was also identified in all treatments, and it was significantly upregulated by RS; however, it was downregulated in the presence of Av compared with its level in the presence of Gl. Cellobiose dehydrogenase (CDH) is extracellularly produced by various lignocellulolytic fungi, and it oxidizes the reducing ends of cellobiose and cello-oligosaccharides to their corresponding 1,5-lactones. In this study, one CDH (gi|66853735) was iTRAQ-quantified in three treatments, and the highest iTRAQ ratio (0.41) was obtained with Av. This ratio was 2.16 times higher than that obtained with Gl.

## Discussion

Most of the renewable carbon on earth exists in the form of lignocellulose, which is mainly composed of cellulose, hemicelluloses and lignin. Fermentable sugars, which are the hydrolyzed products of lignocellulose, are important substrates for biofuel generation [19]. The hydrolysis of lignocellulose to sugars requires a cocktail of lignocellulolytic enzymes. Microbial cellulase and hemicellulase production is dependent on different carbon sources [20]. In this study, several lignocellulases were detected when glucose, Avicel and rice straw were used as carbon sources, and the results indicated that rice straw was the optimal carbon source for the production of lignocellulases, whereas glucose significantly repressed the production of lignocellulases by *A. fumigatus* Z5. Dashtban et al. [21] studied the effect of fourteen different carbon sources on cellulase production by *Hypocrea jecorina* and noted that each strain exhibited minimal cellulase activity when glucose was used as the sole carbon source. In Figure 3A, the Avicel and rice straw lanes had clear new bands at 20 kDa, while it disappeared in the Glucose lane. We checked the LC-MS/MS data, and found some relative proteins around 20 kDa. These proteins included FG-GAP repeat protein (gi|159130639, pI 5.34, theoretical Mw 24.07), endo-1,4-beta-xylanase (gi|66845983, pI 6.27, theoretical Mw 24.33), endoglucanase (gi|70986814, pI 6.02, theoretical Mw 25.79), Cu, Zn superoxide dismutase SOD1 (gi|70997966, pI 5.82, theoretical Mw 16.36), putative protein (gi|159128431, pI 4.7, theoretical Mw 19.27), and all these proteins disappeared in the Glucose lane. Endo-1,4-beta-xylanase and endoglucanase belong to glycoside hydrolases family, and they would be inhibited by glucose. Feedback inhibition of lignocellulases by reducing sugars is very common in most of the filamentous fungi. Chulkin et al. [22] studied CreA, which is considered to be a transcriptional regulator of carbon catabolite repression in *Penicillium canescens*. These authors found that the CreA protein localized to the nucleus irrespective of the nature of the carbon source or the glucose concentration in the medium. *ace1* was also a repressor of cellulase and xylanase. Nina Aro et al. [23] characterized the effect of a deletion of the *ace1* gene in *Trichoderma reesei*, and they found that deletion of *ace1* caused an increase in the expression of all the main cellulase and xylanase genes.

Lignocellulases, especially cellulases and hemicellulases, play major roles during the biodegradation of lignocellulosic biomass, which is considered to be an abundant renewable resource. The production of lignocellulases is the main bottleneck during bioenergy production. In this study, all types of enzymes exhibited maximal activity when rice straw was used as a carbon source. Hideno et al. [24] noted that the choice of carbon source was an important factor in the production of cellulases and hemicellulases. Rice straw has gained considerable interest because of its high annual production (731 million tons), which can be potentially transformed into approximately 205 billion liters of bio-ethanol per year [25]. Rice straw may be suitable for the production of cellulase and hemicellulase due to its physical and chemical characteristics, and cellulose (41%) and hemicellulose (20%) are the main components in rice straw [14].

Hiroki et al. [26] used zymography to detect endoglucanase and xylanase activities. In this study, endoglucanase, exoglucanase, β-glucosidase, xylanase, and pectinase were detected in the gel, and the results revealed that rice straw was a suitable carbon source for the production of lignocellulases by *A. fumigatus* Z5. This finding was consistent with previous studies [27,28]. Asiya et al. [27] reported the differential expression of endoglucanase and β-glucosidase isoforms in *Aspergillus terreus* cultivated with different carbon sources. These authors found that the maximum β-glucosidase activity (28.0 U·g^-1^) and the greatest number of bands were obtained when rice straw was used as the carbon source.

The biodegradation of various types of lignocellulosic biomass into reducing sugars requires a wide range of enzymes including cellulases, hemicellulases, pectinases, peroxidases, and others [29]. The complete transformation of cellulose into sugar requires a series of enzymes including endoglucanases randomly cleaving the cellulose chain, exoglucanases attacking the chain ends, and β-glucosidases catalyzing the hydrolysis of cellobiose into glucose. The efficient hydrolysis of lignocellulosic biomass depends on the appropriate expression of endoglucanase, exoglucanase and β-glucosidase [30], which work synergistically to degrade the cellulose fraction. Thus, a lack (or low expression level) of any of these enzymes would affect the hydrolysis of lignocellulose biomass. *Trichoderma reesei* was considered to be a potential producer of endoglucanases, cellobiohydrolase and β-glucosidase [19]; however, low β-glucosidase activity limited further application of *T. reesei*. Although *Aspergillus niger* has the potential to produce all the cellulase components with high β-glucosidase activity, its cellulose hydrolysis efficiency was limited due to a low level of endoglucanase expression [31]. The main advantages of the *A. fumigatus* Z5 cellulase system were its thermostable characteristics and broad pH range; however, the enzyme activity determination results indicated that its cellobiohydrolase activity (0.59 U·ml^-1^) was low, which would limit its application in cellulose degradation. Co-culturing with a strain which has high levels of cellobiohydrolase is likely to be an effective method for improving cellulose hydrolysis efficiency [32].

In this study, the iTRAQ quantification results with RS showed significant upregulation of all the quantified hemicellulases, indicating the lignocellulosic degradation potential of *A. fumigatus* Z5. However, there was no significant upregulation of the quantified hemicellulases in the presence of Av, which might be due to the characteristics of the substrate. Hemicelluloses embedded in the cell wall of rice straw act as bridges that connect pectin to cellulose, forming cross-linked fibers [29]. Celluloses and pectins are easily accessible to cellulases and pectinase only when hemicelluloses are hydrolyzed by hemicellulases [33]. Therefore, hemicellulases play an important role in the degradation of lignocellulosic biomass. Hemicellulases are enzymes that degrade hemicellulose polymers, and these enzymes include endo-1,4-β-xylanase, R-glucuronidase, β-xylosidase, acetylxylan esterase and R-L-arabinofuranosidase [29]. Pectin is a complex heteropolymer of homogalacturonan, which can be converted into reducing sugars and subsequently fermented into bioethanol [34]. Pectin can be hydrolyzed by pectate lyase, which is responsible for the eliminative cleavage of pectate [35]. In this study, three and four pectate lyases were identified by LC-MS/MS in the presence of Av and Rs, respectively, while none were identified with Gl. Manavalan et al. [36] revealed that most pectinases can be induced by cellulose. However, the iTRAQ results showed that pectate lyase A (gi|66846844) was detected in all treatments, thus revealing that pectate lyase A is a constitutively expressed enzyme. On the other hand, these results indicate that iTRAQ technology is more accurate and sensitive than common LC-MS/MS.

## Conclusions

The evaluation of various lignocellulase activities and zymogram analysis results indicated that *A. fumigatus* Z5 has great potential for use in the bioenergy industry, and the production of various microbial cellulases and hemicellulases was dependent on the carbon source. Proteomics is a very effective technique for understanding the mechanism by which different lignocellulolytic enzymes are secreted by *A. fumigatus* Z5. The cultivation of *A. fumigatus* Z5 with different lignocellulosic substrates (rice straw and Avicel) highlighted the differential expression levels of lignocellulolytic enzymes including cellulases, hemicellulases, glycoside hydrolases, chitinases, esterases, peptidases, lipases, protein translocating transporters and hypothetical proteins.

### Experimental procedures

### Strain growth conditions and protein extraction

The lignocellulosic decomposing strain *A. fumigatu*s Z5 was isolated from the compost and identified as previously reported [13]. The strain was grown on potato dextrose agar (PDA) medium conidia production, and then conidia were harvest by washing the plate with 10 ml sterile ddH_2_O, after removing of the mycelia, the conidia were re-suspended and adjusted the concentration to 1×10^6^ conidia ml^-1^. 1% (v/v, ml suspension per ml liquid medium) fresh conidia suspension of strain Z5 was inoculated into nine separate test flasks (1L) containing Mandels’ salts solution [37] supplemented with 1% (w/v) different carbon sources (rice straw, Avicel and glucose), and all the test flasks were incubated at 50°C and 170 rpm. After 7 days of incubation, the substrates and fungal biomass were removed by centrifugation (10,000 g for 10 min at 4°C) and further clarified by filtration through a 0.45 μm membrane (Beyotime, China). The clear supernatant was used as the crude enzyme extract in the subsequent experiments. The fungal biomass was collected by filtrated through two layers of gauze, and then washed by ddH_2_O for three times. The dry weights of different samples were obtained by determination the constant weight at 105°C. The proteins in the clear supernatant were concentrated by lyophilization and redissolved in acetate buffer (50 mM, pH 5.0).

### Enzyme assays

Endoglucanase (EG) activity was estimated by the DNS method [38] with glucose as a standard. Substrate solution for the EG assays consisted of 1% CMC (Sigma, USA) in sodium acetate buffer (50 mM, pH 5.0). 0.1 ml crude enzyme was mixed with 2 ml substrate solution and 2.9 ml distilled water, and incubated for 20 min at 50°C. One unit of enzyme activity was defined as the amount of enzyme required to release 1 μmol of reducing sugars in 1 min. Cellobiohydrolase (CBH) activity and β-glucosidase activity were measured by a microtiter plate method using the chromogenic substrates β-nitrophenyl-β-D-cellobioside (pNPC) (Sigma, USA) [39] and β-nitrophenyl-β-D-glucopyranoside (pNPG) (Sigma, USA) [40], respectively. 10 μl crude enzyme was mixed with 25 μl of 200 mM sodium acetate buffer (pH 5.0), 25 μl 10 mM substrate and 40μl of distilled water. The plate was incubated at 50°C for 10 min, the reaction was terminated by adding 100μl 1M Na_2_CO_3_ and the colour developed was read at 405 nm. One unit of enzyme activity was defined as the amount of enzyme required to release 1 μmol of pNP per minute under the above assay conditions.

Activities of the hemicellulose enzymes, including laminarinase (endo-β-1,3-glucanase), lichenase (endo-β-1,3;1,4-glucanase), and xylanase (endo-β-1,4-xylanase and 1,4-β-D-xylan xylanohydrolase), were measured according to Linton et al. [8], and laminarin (Aladdin Chemistry Co. Ltd, Cat. No. 9008-22-4), lichenin (Megazyme, Bry, Ireland) and xylan (from birchwood; Sigma, CAS: 9014-63-5) were used as substrates, respectively. 10 μl crude enzyme was incubated with 100 μl 1% (w/v) substrate and 90 ml of 0.1mol L^-1^ Na acetate buffer (pH 5.5) at 50°C for 10 min, and the reaction was stopped with 50 ml of 0.3 mol L^-1^ HCl and the mixture was then neutralized with 10 ml of 2.5 mol L^-1^ K_2_CO_3_. One unit of enzyme activity was defined as the amount of enzyme required to release 1 μmol of reducing sugars from the substrate in 1 min.

Pectin lyase activity was determined by monitoring the increase in absorbance at 235 nm according to Delgado et al. [41]. The reaction mixture contained 1.0 ml of 1.0% pectin, 1.0 ml of 0.05 M Tris-acetate buffer (pH 8.8) and 0.5 ml culture filtrate and was incubated at 40°C for 20 min. One 0.5-ml aliquot was taken from the reaction mixture and added to a test tube containing 4.5 ml of 0.01 M HC1 to stop the reaction. One unit of pectin lyase activity was defined as the amount of enzyme that produced an increase in absorbance of 0.1 at 235 nm in the reaction mixture under the assay conditions. The assay of polygalacturonase activity was followed Pathak and Sanwal [42]. The assay reagents contained 0.2 ml of 0.2 M acetate buffer (pH 4.5), 0.3 ml of 1% polygalacturonic acid in 0.05 M acetate buffer solution (pH 4.5) and 0.5 ml crude enzyme, and this reaction system was incubated for 20 min at 40°C. One unit of polygalacturonase was defined by the catalyzation of the hydrolytic cleavage to form 1 μM of galacturonic acid in 1min under standard conditions.

### Protein assay, SDS-PAGE and zymogram analysis

Protein content was quantified in the supernatants using a Micro BCA protein assay kit (Beyotime, China) according to the manufacturer’s protocol. The developed color was read at 562 nm using a Multi-Detection Microplate Reader (Spectra max M5, Molecular Devices, Sunnyvale, CA), and bovine serum albumin (BSA) was used as the standard. SDS-PAGE was performed using a 10% (w/v) polyacrylamide gel as described by Laemmli [43]. Based on the protein concentration, 40 μl supernatants (RS:47.5 μg, Av: 21.0 μg, Gl:12.8 μg) from each treatment were loaded on the gel. After destaining, the gel was analyzed using Quantity one (Bio-Rad).

Zymogram analysis of endoglucanase was performed by adding CMC to the gel [44]. Detection of the in-gel β-glucosidase activity was performed according to Kim et al. [45] with some modifications. After renaturation, the gels were washed with distilled water and subsequently overlaid with 0.5 mM 4-methylumbelliferyl β-D-glucopyranoside (Sigma, USA) in 0.1 M succinate buffer (pH 5.8), and the presence of a fluorescent reaction product was determined under UV light (365 nm) after incubating the gels at 50°C for 5 min. Detection of the in-gel exoglucanase activity performed similarly to that of β-glucosidase activity, but the substrate was 4-methylumbelliferyl-β-D-cellobioside (Sigma, USA).

Zymogram analysis of xylanase was performed by the method of Tseng et al. [46]. Approximately 10 μg of each sample was subjected to SDS-PAGE using a gel containing 0.5% xylan. After renaturation, the gel was stained with a 0.1% (w/v) Congo red solution for 30 min before destaining with l M NaCl. In-gel pectinase activity was detected following the method of Schneider et al. [47]. 0.1% pectin was incorporated into the gel, and the proteins were renatured after electrophoresis. The gels containing pectin were stained with 0.05% ruthenium red for 10 min and subsequently washed with water until colorless bands could be detected against a purple background.

### SDS-PAGE (1D) coupled to LC-MS/MS

Protein digestion and peptide extraction were performed according to Liu et al. [48] with some modifications. After SDS-PAGE analysis, the bands sliced from different lanes were washed with ddH_2_O twice and de-stained at room temperature with de-staining solution (25mM NH_4_HCO_3_、50%ACN) for 30 min, and then kept in dehydration solution 1 (50% ACN) for 30 min. The bands were treated with reduction solution 1 (25mM NH_4_HCO_3_、1mM DTT) at 57°C for 1 h after washing by dehydration solution 2 (100% ACN) for 30 min, and then transferred to reduction solution 2 (25mM NH_4_HCO_3_、50mM IAA) and kept at room temperature for 30 min. The imbibition solution (25mM NH_4_HCO_3_) was added after remove the reduction solution 2. After that, the gels were treated with dehydration solution 1 and dehydration solution 2 each for 30 min in order. The gels were rehydrated in 10 μl digest solution (0.02 ug ul^-1^ trypsin in cover solution) for 30 min, and 20 μl cover solution (25mM NH_4_HCO_3_, 10% CAN) was added for digestion 16 hours at 37°C. The supernatants were transferred into another tube. The gels were extracted once with 50 μl extraction buffer (5% TFA、67%ACN) at 37°C for 30min. The peptide extracts and the gel supernatant were combined and then completely dried. Mass spectrometric analysis was carried out as follows: The samples were resuspended with redissolved solution, and chromatography was performed using an Eksigent nanoLC-Ultra™ 2D System (AB SCIEX). The samples were loaded on an in-house packed trap column (100 μm × 3 cm, C18, 3 μm 150 Å) and washed for 10 min at 4 μl min^-1^. Subsequently, an elution gradient of 5-35% acetonitrile (0.1% formic acid) over 70 min was run with an in-house packed analytical column (75 μm × 15 cm, C18, 3 μm 150 Å) with a spray tip. Data acquisition was performed with a TripleTOF 5600 System (AB SCIEX), and the data were acquired using an ion spray voltage of 2.5 kV. The MS was operated with TOF-MS scans. For IDA, survey scans were acquired in 250 ms. The data were processed with ProteinPilot software v. 4.0 (AB SCIEX). The tolerances were specified as ± 0.05 Da for peptides and ± 0.05 Da for MS/MS fragments. False discovery rate (FDR) analysis was also performed using the integrated tools in ProteinPilot.

### iTRAQ labeling and automated 2D LC-MS/MS protein identification

One gram solid secretome powder of *A. fumigatus* Z5 from different treatment was dissolved in 3 ml ddH_2_O and concentrated by running through an Amicon YM-3 centrifugal filter unit with a 3-kDa molecular mass cut-off membrane filter (Millipore, Bedford, MA). After quantified by BCA method, 50 μg (RS), 22.1 μg (Av) and 13.5 μg (Gl) proteins were used for protein digestion and iTRAQ labeling. The protein digestion was performed following the method of Riviere et al. [49]. iTRAQ labeling was carried out in accordance with Adav et al. [50,51], and the peptide samples were labeled using the iTRAQ Reagent Multiplex Kit (Applied Bio systems, Foster City, CA) according to the manufacturer’s protocol. The samples from RS, Av and Gl substrates were labeled with the iTRAQ tags 116, 117 and 119, respectively.

Automated 2D LC-MS/MS analysis was carried out according to Liu et al. [48] with some modifications. The labeled peptides were desalted through a C18 solid phase extraction column (Waters Corporation, Milford, USA) and subsequently dried using a vacuum centrifuge. All the extracted peptides were resuspended in loading buffer (5 mM ammonium formate containing 5% acetonitrile, pH 3.0). The collected samples were separated and analyzed on the 2D-nano LC/MS system. The LTQ Orbitrap XL mass spectrometer (Thermo Electron Corp.) was operated in the data-dependent mode to switch automatically between MS and MS/MS acquisition [52]. During MS/MS, the activating parameters of the precursor ions were 40% normalized collision energy and an activation time of 30 ms. The relative abundance of the peptides and proteins in different samples were reflected by the peak intensities of the three iTRAQ reporter ions.

Protein identification and quantification for the iTRAQ experiments were performed with ProteinPilot software (version 4.0; Applied Biosystems, USA). The database used for searching was the *A. fumigatus* Af293 NCBI database (with 19983 entries). The Paragon Algorithm in ProteinPilot was used for peptide identification and isoform-specific quantification. The data search parameters were set up as follows: Trypsin (KR) cleavage with two missed cleavage sites was considered along with fixed modification of cysteines by methyl methanethiosulfonate (MMTS). iTRAQ modification of peptide/protein identification and quantification was also performed with the ProteinPilot software version 4.0. The database and data search parameters were the same as above. iTRAQ modification of lysine residues, peptide N termini, and methionine oxidation were set as variable modifications. A strict cutoff for protein identification was applied with an unused ProtScore ≥1.3, which corresponds to a confidence limit of 95%, to minimize false positive results. At least two peptides with 95% confidence were considered for protein quantification. The resulting data set was auto bias-corrected to eliminate any variation due to unequal mixing when combining different labeled samples. For iTRAQ quantitation, the peptide used for quantification was automatically selected by the Pro Group algorithm (at least two peptides with 99% confidence) to calculate the reporter peak area, error factor (EF), and p-value. N termini, methionine oxidation and iTRAQ modification of lysine residues were set as variable modifications. To minimize false positive results, a strict cut off for protein identification was applied with an unused ProtScore ≥1.3, which corresponds to a confidence limit of 95%, and at least two peptides with 95% confidence were considered for protein quantification. The resulting data set was auto bias-corrected to eliminate any variation due to unequal mixing when combining different labeled samples. For iTRAQ quantitation, the peptide used for quantification was automatically selected by the Pro Group algorithm (at least two peptides with 99% confidence) to calculate the reporter peak area, error factor (EF), and p-value. The existence of signal peptide sequences was determined using the signal peptide prediction program SignalP, version 4.1 (http://www.cbs.dtu.dk/services/SignalP/).

## Abbreviations

EG: Endoglucanase; CBH: Cellobiohydrolase; pNPC: β-nitrophenyl-β-D-cellobioside; pNPG: β-nitrophenyl-β-D-glucopyranoside; 4-MUG: 4-methylumbelliferyl β-D-glucopyranoside; 4-MUC: 4-methylumbelliferyl-β-D-cellobioside.

## Competing interests

The authors declare that they have no competing interests.

## Authors’ contributions

DL performed the majority of the experiments and wrote the manuscript. JL extracted the secretomes. MW and YM performed the LC-MS/MS experiments and data analyses. YS determined the lignocellulase activities, and SZ performed the zymography analyses. RZ assisted in the drafting and revision of the manuscript. QS was the corresponding author, and he supervised the work and contributed to the manuscript. All authors have read the manuscript and approved the submission to this journal.

## Supplementary Material

Additional file 1: Figure S1 Ligninolytic enzyme and cellobiose dehydrogenase activities in the secretome of *A. fumigatus* Z5 in the presence of different carbon sources. The results are presented as the mean of three replicates, and bars indicate the standard error of three replicates. Time course profiles of ligninolytic enzymes (i.e., laccase, manganese peroxidase and lignin peroxidase) production by *A. fumigatus* Z5 on different carbon sources are shown in A, B and C, respectively. The production of cellobiose dehydrogenase (CDH) by *A. fumigatus* Z5 in the presence of different carbon sources is described in D. Click here for file

Additional file 2 The LC-MS/MS identification results. Click here for file

Additional file 3: Table S1 The set of 35 proteins common identified in all three treatments i.e. RS, Av and Gl. Click here for file

Additional file 4 The iTRAQ quantification results. Click here for file
