# Supplementary figures and images for: Secretome diversity and quantitative analysis of cellulolytic Aspergillus fumigatus Z5 in the presence of different carbon sources
Source: Biotechnol Biofuels. 2013 Oct 16;6:149. doi: 10.1186/1754-6834-6-149 (PMC3853031; doi:10.1186/1754-6834-6-149)

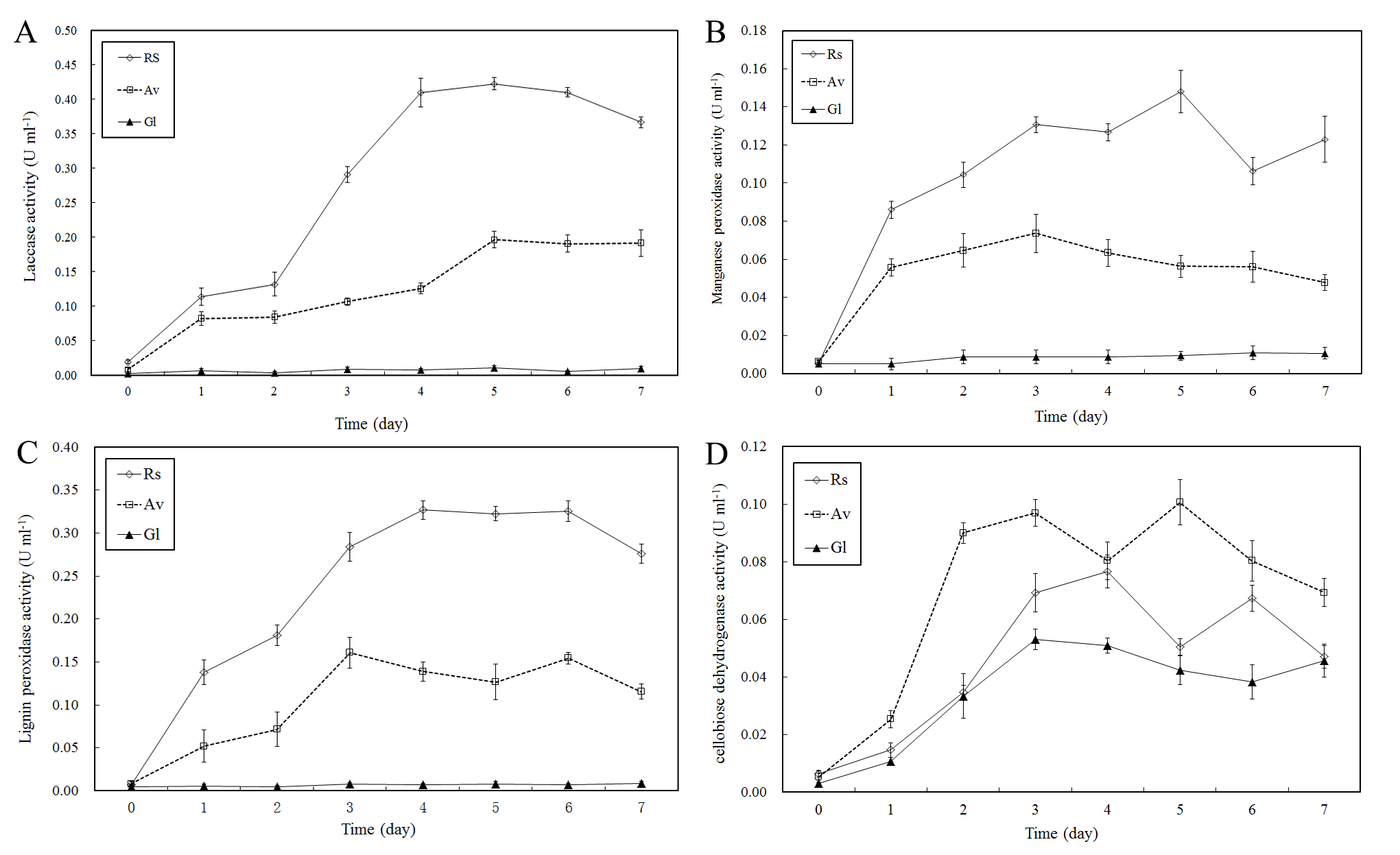

Supplement: Additional file 1: Figure S1 — Ligninolytic enzyme and cellobiose dehydrogenase activities in the secretome of A. fumigatus Z5 in the presence of different carbon sources. The results are presented as the mean of three replicates, and bars indicate the standard error of three replicates. Time course profiles of ligninolytic enzymes (i.e., laccase, manganese peroxidase and lignin peroxidase) production by A. fumigatus Z5 on different carbon sources are shown in A, B and C, respectively. The production of cellobiose dehydrogenase (CDH) by A. fumigatus Z5 in the presence of different carbon sources is described in D. [file 1754-6834-6-149-S1.tiff]
