# Supplementary material for: Secretome diversity and quantitative analysis of cellulolytic Aspergillus fumigatus Z5 in the presence of different carbon sources
Source: Biotechnol Biofuels. 2013 Oct 16;6:149. doi: 10.1186/1754-6834-6-149 (PMC3853031; doi:10.1186/1754-6834-6-149)
Supplement: Additional file 3: Table S1 — The set of 35 proteins common identified in all three treatments i.e. RS, Av and Gl. [file 1754-6834-6-149-S3.docx]

Additional Table 1 The proteins common identified in all three treatments i.e. RS, Av and Gl

| Accession No. | Protein Name | pI | Mw | Family | Signal Peptides |
| --- | --- | --- | --- | --- | --- |
| gi\|129555482 | cytochrome P450 | 8.52 | 60.14 | [p450](http://pfam.sanger.ac.uk/family/PF00067.17) (PF00067) | N |
| gi\|129557040 | Ser/Thr protein phosphatase family protein | 6.53 | 40.47 | Metallophos (PF00149) | N |
| gi\|129558344 | class V chitinase | 5.21 | 46.49 | GH 18 (PF00704) | Y |
| gi\|129558573 | esterase | 8.51 | 28.77 | **－** | Y |
| gi\|146324637 | hybrid PKS-NRPS enzyme | 5.71 | 435.73 | Acyl_transf_1 (PF00698) | N |
| gi\|159122696 | extracellular arabinanase | 5.35 | 45.02 | GH43 (PF04616) | N |
| gi\|66843951 | endo-chitosanase | 6.22 | 25.19 | GH75 (PF07335) | Y |
| gi\|66844481 | high affinity methionine permease | 9.13 | 60.03 | AA_permease2 ( PF13520) | N |
| gi\|66844580 | glycosyl hydrolase family 43 protein | 5.57 | 49.67 | GH 43 (PF04616) | Y |
| gi\|66845136 | beta-1,6-glucanase Neg1 | 5.51 | 51.43 | GH 30 (PF02055) | Y |
| gi\|66845449 | alpha-galactosidase | 5.38 | 56.33 | Melibiase (PF02065) | N |
| gi\|66845766 | extracellular lipase | 5.59 | 31.44 | Lipase 3 (PF01764) | Y |
| gi\|66845983 | endo-1,4-beta-xylanase (XlnA) | 6.27 | 24.49 | GH 11 (PF00457) | Y |
| gi\|66846140 | cellobiohydrolase | 5.08 | 47.80 | GH 6 (PF01341) | Y |
| gi\|66846336 | endo-1,4-beta-glucanase | 5.28 | 35.53 | GH 61 (PF03443) | Y |
| gi\|66846526 | beta-D-glucoside glucohydrolase | 5.65 | 78.38 | GH 3 (PF00933) | Y |
| gi\|66846833 | arabinosidase | 5.82 | 52.56 | GH 43 (PF04616) | N |
| gi\|66846837 | glucan 1,4-alpha-glucosidase | 5.04 | 67.10 | GH 15 (PF00723) | Y |
| gi\|66846860 | extracellular glycosyl hydrolase/cellulase | 5.92 | 42.92 | GH 62 (PF03664) | Y |
| gi\|66846861 | xylosidase/glycosyl hydrolase | 5.17 | 55.65 | GH 43 (PF04616) | Y |
| gi\|66847207 | HECT domain protein | 8.44 | 151.67 | HECT (PF00632) | N |
| gi\|66847547 | C6 finger domain protein C6 | 9.26 | 46.06 | Zn_clus (PF00172) | N |
| gi\|66848496 | thioredoxin reductase GliT | 5.44 | 36.00 | Pyr_redox_2 (PF07992) | N |
| gi\|66848870 | endo-1,4-beta-xylanase | 5.48 | 42.14 | GH 10 (PF00331) | Y |
| gi\|66848977 | telomere-associated RecQ helicase | 8.64 | 163.57 | [DUF3505](http://pfam.sanger.ac.uk/family/PF12013.3) (PF12013) | N |
| gi\|66849090 | penicillolysin/deuterolysin metalloprotease | 5.71 | 39.40 | Peptidase_M35 (MF02102) | Y |
| gi\|66850620 | extracellular cell wall glucanase Crf1 | 4.6 | 40.28 | GH 16 (PF00722) | Y |
| gi\|66851740 | endo-1,4-beta-xylanase | 5.26 | 39.90 | GH 10 (PF00331) | Y |
| gi\|66851935 | aldose 1-epimerase | 5.62 | 50.73 | *Aldose-epim* (PF01263) | N |
| gi\|66852539 | glutaminase GtaA | 4.71 | 76.15 | DUF1793 (PF08760) | Y |
| gi\|66853400 | translation initiation factor eIF-2B subunit family protein | 6.28 | 64.34 | *W2* (PF02020) | N |
| gi\|70981394 | extracellular arabinanase | 5.35 | 44.99 | GH 43 (PF04616) | Y |
| gi\|70982865 | MFS transporter | 7.21 | 48.48 | MFS_1 (PF07690) | N |
| gi\|70986104 | mycelial catalase Cat1 | 5.5 | 79.91 | Catalase (PF00199) | N |
| gi\|70997966 | Cu,Zn superoxide dismutase SOD1 | 5.82 | 16.36 | Sod_Cu (PF00080) | N |

* The existence of signal peptide sequences was determined using the signal peptide prediction program SignalP, version 4.1 (<http://www.cbs.dtu.dk/services/SignalP/>). “**－**” means not detected,

“Y” means with signal peptides, “N” means without signal peptides; * All the proteins were divided into different family, and the Pfam numbers were provided by Pfam 27.0 (http://pfam.sanger.ac.uk/) ;

* pI means “isoelectric point”, Mw means “molecular weight” , and both two parameters were theoretical values speculated by Compute pI/Mw tool (http://ca.expasy.org/tools/pi_tool.html).
